# The use of montelukast for pruritus in neurofibromatosis 1

**DOI:** 10.1016/j.jdcr.2022.05.022

**Published:** 2022-06-02

**Authors:** Nicole L. Edmonds, Lydia A. Luu, Darren J. Guffey

**Affiliations:** a University of Virginia School of Medicine, Charlottesville, Virginia; b Department of Dermatology, University of Virginia, Charlottesville, Virginia

**Keywords:** general dermatology, mast cell, medical dermatology, montelukast, neurofibroma, neurofibromatosis, NF1, pheochromocytoma, sunitinib, NF1, neurofibromatosis 1, RTK, receptor tyrosine kinases

## Introduction

Neurofibromatosis 1 (NF1) is caused by an autosomal dominant mutation in the NF1 tumor suppressor gene, which predisposes individuals to various neuronal, hematopoietic, and skeletal disorders.^1^ The major diagnostic features include café-au-lait patches, skin-fold freckling, iris lisch nodules, optic pathway glioma, and bony dysplasia, as well as cutaneous, subcutaneous, and plexiform neurofibromas, which are pathognomonic for the disease.^1^ NF1 patients also have a higher predilection for developing tumors, such as pheochromocytomas, gastrointestinal stromal tumors, and paraganglionomas.^2^ Pheochromocytomas, in particular, occur in only 0.1%–5.7% of patients with neurofibromatosis.^2^ Therefore, there is minimal literature on the treatment and therapeutic side effects of pheochromocytomas in the context of NF1. Here, we present a case of a patient with NF1 and a pheochromocytoma treated with sunitinib who subsequently developed pruritus of her neurofibromas. We present this case in order to describe this rare side effect of sunitinib in NF1 patients and offer a new possible treatment approach.

## Case report

**Fig 1:**
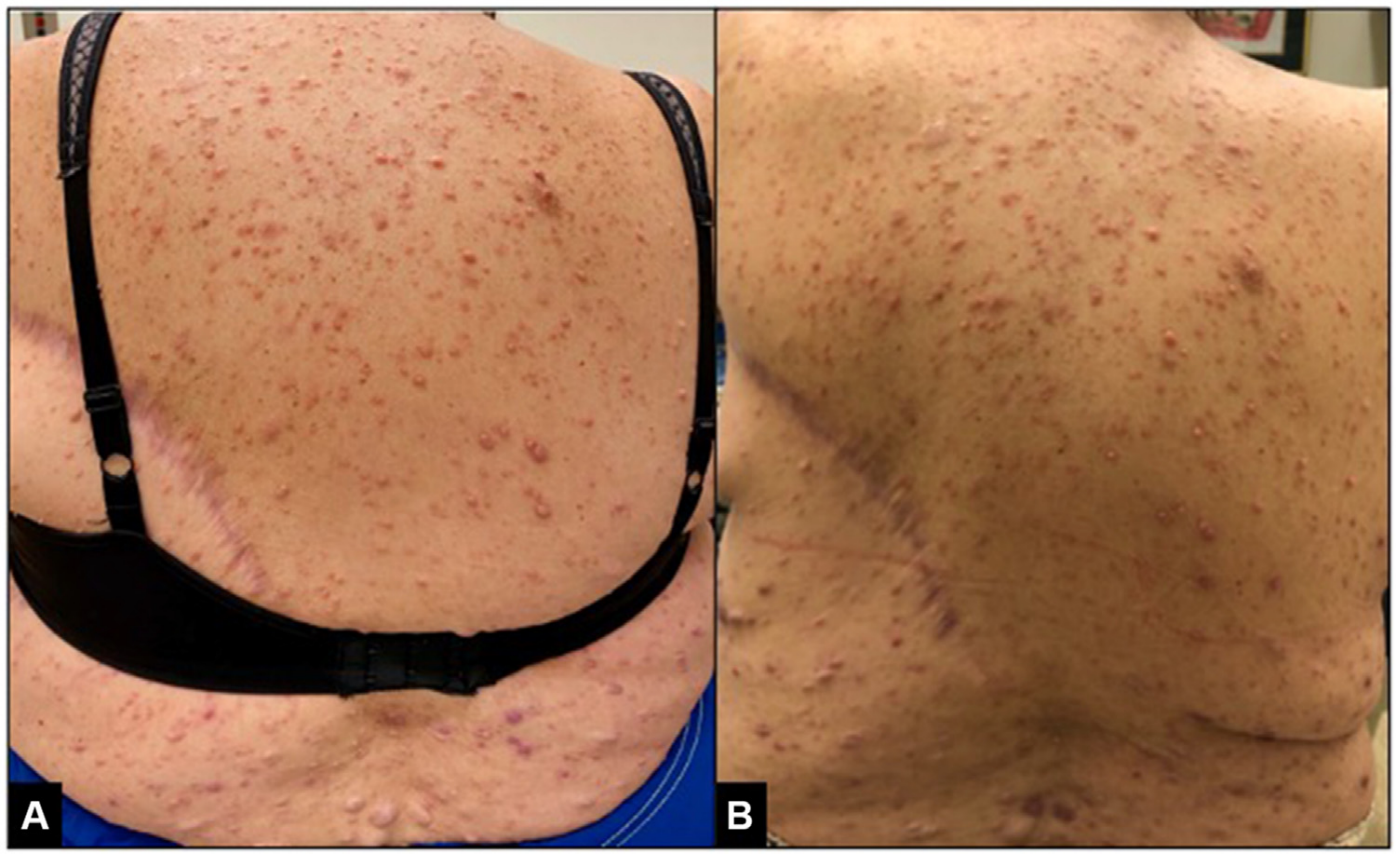
Pink, inflamed neurofibromas narrowly surrounded by small, pink plaques were observed, most notably on the upper back before therapy with montelukast (**A**). A significant reduction in neurofibroma inflammation was observed after therapy with montelukast (**B**).

A 59-year-old woman with a past medical history of NF1 complicated by multiple malignancies, including metastatic malignant pheochromocytoma, presented to the dermatology clinic with new inflammation and pruritus of her neurofibromas. The patient reported that 1 month prior, she had begun therapy with sunitinib for her metastatic pheochromocytoma. She noticed a reduction in size of her cutaneous neurofibromas with this new therapy; however, she also experienced new inflammation and pruritus of the neurofibromas. On examination, she was found to have innumerable soft, pink-to-skin–colored papules throughout her trunk and extremities that appeared inflamed (Fig 1, *A*). An excisional biopsy was performed of an 11 mm nodule with an overlying hemorrhagic crust on her left deltoid in order to rule out a malignant peripheral-nerve-sheath tumor, but pathology revealed a benign, traumatized neurofibroma. Given the fact that neurofibromas contain copious mast cells, her pruritus was treated with an agent that inhibits mast cell degranulation, montelukast. After 1 month on 10 mg montelukast daily, the patient reported drastic improvement of the inflammation and pruritus of her neurofibromas (Fig 1, *B*) without any additional side effects. The patient has remained on montelukast for 9 months with sustained improvement.

## Discussion

NF1 encodes the protein neurofibromin, which regulates p21ras activation and its downstream effects.^3^ Attempts to target p21ras itself have been challenging; therefore, current therapy approaches focus on targeting the receptor tyrosine kinases (RTK) that activate p21ras.^3^ Sunitinib is a highly selective RTK inhibitor with activity against *c-kit*, *PDGFR, FGFR, RET*, and *VEGFR*, which are all pathways that have been implicated in the pathogenesis of both pheochromocytomas and neurofibromas.^3^ Much of the literature to date has studied the effect of sunitinib on pheochromocytomas and plexiform neurofibromas, which have malignant potential.^4^ Nevertheless, cutaneous neurofibroma formation arises from similar biochemical pathways as pheochromocytomas and plexiform neurofibromas and are therefore also affected by therapy with sunitinib. Considering that many patients with plexiform neurofibromas or NF1-associated pheochromocytomas also have cutaneous and subcutaneous neurofibromas, the effect of sunitinib on all types of neurofibromas is worth evaluating. In this case, the patient was treated with sunitinib for her pheochromocytoma and concomitantly experienced reduction in size of her cutaneous neurofibromas.

In addition to the reduction in size of her neurofibromas with sunitinib therapy, the patient also experienced significant pruritus of her neurofibromas. Though adverse skin reactions occur in approximately 90% of cases treated with angiogenic inhibitors,^5^ pruritus is not a commonly reported side effect of sunitinib.^6^ Pruritus has been reported in studies of sorafenib and imatinib, which are also RTK inhibitors; however, the cause of this pruritus is unknown, and treatment with antihistamines, steroids, and topical anesthetics has demonstrated only a variable response.^7^ No studies have evaluated the cause of RTK inhibitor induced pruritus within neurofibromas. Nevertheless, it is known that sunitinib has a direct effect on mast cells and that mast cells play a critical role in neurofibroma tumorigenesis and pruritus.^3^^,^^8^ Therefore, we hypothesized that a leukotriene receptor antagonist, which has downstream effects on mast cell degranulation,^9^ may be an effective therapy for patients with sunitinib-induced pruritus within their neurofibromas. The patient was started on montelukast monotherapy and experienced relief of her pruritus within 1 month of treatment without any side effects. To the authors’ knowledge, the use of leukotriene receptor antagonists has never been reported in this setting. We present this case in order to suggest a potential new treatment approach for this nuanced condition.

## Conflicts of interest

None disclosed.

## References

[1] Ferner R.E. (2007). Neurofibromatosis 1. Eur J Hum Genet.

[2] Hari Kumar K.V., Shaikh A., Sandhu A.S., Prusty P. (2011). Neurofibromatosis 1 with pheochromocytoma. Indian J Endocrinol Metab.

[3] Ferguson M.J., Rhodes S.D., Jiang L. (2016). Preclinical evidence for the use of sunitinib malate in the treatment of plexiform neurofibromas. Pediatr Blood Cancer.

[4] Baudin E., Goichot B., Berruti A. (2021). 567O First international randomized study in malignant progressive pheochromocytoma and paragangliomas (FIRSTMAPPP): an academic double-blind trial investigating sunitinib. Ann Oncol.

[5] Ara M., Pastushenko E. (2014). Antiangiogenic agents and the skin: cutaneous adverse effects of sorafenib, sunitinib, and bevacizumab. Actas Dermosifiliogr.

[6] Vignand-Courtin C., Martin C., Le Beller C., Mateus C., Barbault-Foucher S., Rieutord A. (2012). Cutaneous side effects associated with sunitinib: an analysis of 8 cases. Int J Clin Pharm.

[7] Wu J., Lacouture M.E. (2018). Pruritus associated with targeted anticancer therapies and their management. Dermatol Clin.

[8] Staser K., Yang F.C., Clapp D.W. (2010). Mast cells and the neurofibroma microenvironment. Blood.

[9] Cikler E., Ersoy Y., Cetinel S., Ercan F. (2009). The leukotriene d4 receptor antagonist, montelukast, inhibits mast cell degranulation in the dermis induced by water avoidance stress. Acta Histochem.

